# Brief admission by self-referral for individuals with self-harm and suicidal ideation: a qualitative study based on focus groups exploring relatives’ experiences

**DOI:** 10.1080/17482631.2024.2353460

**Published:** 2024-05-13

**Authors:** Rose-Marie Lindkvist, Joachim Eckerström, Kajsa Landgren, Sofie Westling

**Affiliations:** a Department of Clinical Sciences Malmö, Psychiatry, Lund University, Lund, Sweden; b Centre for Psychiatry Research, Department of Clinical Neuroscience, Karolinska Institutet and Stockholm Health Care Services, Region Stockholm, Stockholm, Sweden; c Department of Neurobiology, Care Sciences and Society, Division of Nursing, Karolinska Institutet, Stockholm, Sweden; d Office for Psychiatry and Habilitation, Psychiatric Clinic Lund, Region Skåne, Lund, Sweden

**Keywords:** Self-harm, suicidal ideation, brief admission, relatives, prevention, reflexive thematic analysis, psychiatric inpatient care

## Abstract

**Purpose:**

Brief Admission by self-referral (BA) is a standardized crisis-management intervention for individuals with self-harm and risk for suicide. This study explored relatives’ experiences of BA. Relatives’ perspectives may contribute to an increased understanding of the effects of BA given the relatives’ role as support and informal caregivers as well as being co-sufferers.

**Methods:**

Fourteen relatives to adults with access to BA within one Swedish region participated in focus groups analysed with reflexive thematic analysis.

**Results:**

We generated themes evolving around three meaning-based concepts: access (A low threshold to a safe back-up is crucial and obstacles may easily break faith), independence (Trust in their ability with care and respect), and recovery (The rest and relational recovery we all get are needed and invaluable).

**Conclusions:**

BA brings considerable value to users and relatives, by supporting them to take care of themselves and each other. Communication and involvement of relatives may enhance users’ ability to overcome obstacles to accessing BA. Implementation and adherence may be strengthened by supervision of BA staff and education of emergency care staff. Resources are needed to improve access. Mapping hurdles to BA, support through peers and targeted psychoeducation may improve recovery for BA users and their relatives.

## Introduction

Brief Admission by self-referral (BA) is a standardized crisis management intervention targeting persons with symptoms of borderline personality disorder (BPD), including self-harm and suicidal ideation (Liljedahl, Helleman, Daukantaitė, Westling, et al., 2017). BA is focused on prevention through increased autonomy and self-care based on access to structured and brief self-referrals to hospital. During BA (lasting up to three days and available up to three times per month) users are offered daily supportive meetings with nursing staff, may take part in activities at the ward and are offered meals. As a result of a randomized controlled trial, BA is since 2019 continuously offered to adults with self-harm at risk for suicide in the region Skåne in Sweden (Liljedahl, Helleman, Daukantaité, Westling, et al., 2017; Liljedahl, Helleman, Daukantaitė, Westling, et al., 2017; Westling et al., 2019).

Family or other relatives, partners, neighbours, friends and any significant other (hereafter referred to as *relatives* according to the definition used by the Swedish National Board of Health and Welfare (Socialstyrelsen, 2014) close to those suffering from recurrent self-harm and suicidal ideation, play an essential role in times of crisis. Relatives may beneficially contribute to motivation to seek help as well as effects of provided care, and are therefore according to guidelines recommended to be included and considered in health care (NICE, 2011; Westling et al., 2016). Relatives’ role as social support in times of crisis, as well as being co-sufferers, may induce feelings of guilt, shame, stress, anxiety, and pain, related to self-harm and suicide attempts (Ferrey et al., 2016). Therefore, it is important to evaluate new models of care from the perspective of relatives. This should also be considered in relation to the fact that symptoms of BPD may lead to high levels of conflict and instability in close relationships (Beeney et al., 2018). A recent systematic review points to suicidal behaviour as inducing anxiety among relatives. According to the review, relatives to those suffering from suicidal behaviour perceived access to timely professional support as most effective (Marshall et al., 2023), which is the very aim of BA.

Previous qualitative interview studies on the experiences of BA have mainly focused on the perspectives of patients (Eckerström et al., 2020; Helleman et al., 2018; Lindkvist, Westling, Eberhard, et al., 2021; Lindkvist, Westling, Liljedahl, et al., 2021) and healthcare professionals (Eckerström et al., 2019; Lindgren et al., 2023; Lindkvist et al., 2019). Both perspectives indicated, among other things, that BA, through provision of predictable and easy access to help when approaching crisis, has the potential to reduce the strain on and support the maintenance of important social relations. Being able to self-refer to BA has been described as reducing the burden on relatives, which in turn was described as essential to be able to focus on own recovery. Prior studies among relatives to individuals with access to different versions of psychiatric self-referral models have supported these notions of providing a breathing space for relatives, reducing their burden, and giving a sense of daring to let go (Hultsjö, Rosenlund, et al., 2023; Socialstyrelsen 2021). Relatives may offer valuable input for maintaining and enhancing BA as a crisis management tool. Overall, there is an increasing awareness of the value of taking the perspective of relatives, given that informal care is a cornerstone in many health care systems (Zigante, 2018). According to recent standards developed by the Swedish National Board of Health and Welfare the perspective of relatives should be integrated into health care, as such an approach will improve conditions for providing the right care, benefit relatives and reduce costs (Socialstyrelsen, 2023).

With the present study we aim to complement previous research by analysing experiences of relatives to adults with access to BA in Skåne, Sweden. The research questions were as follows: What are the experiences of BA among relatives to adult individuals with self-harm and risk for suicide? What are relatives’ suggestions for improving and adjusting BA for individuals with self-harm and risk for suicide?

## Materials and methods

### Design

In this study we applied a descriptive inductive research approach. We performed qualitative data collection through focus groups (Krueger & Casey, 2015) which we analysed with reflexive thematic analysis (Braun & Clarke, 2006, 2019, 2021, 2023).

### Participants and setting

We included relatives of individuals who had access to BA in Skåne, Sweden, a region with 1.4 million inhabitants. BA was available at four units (Lund, Malmö, Helsingborg, and Kristianstad). The organization of BA was adapted to local conditions and available hospital premises. In Lund BA was provided in a separate unit for BA with eight single rooms. In Malmö and Kristianstad, BA was located at units which also provided emergency care admissions, including compulsory care (hereafter referred to as mixed units). These units contained both single and shared rooms. In Helsingborg there were four separate rooms for BA situated in a separate corridor reached by passing through a psychiatric inpatient emergency care unit. To recruit potential study participants, we sent letters to all adults with an active BA contract in the Skåne region in May to September 2023 (see Table 1). These letters contained information about the study and encouraged recipients to pass the details to their relatives.

**Table 1.** Letters per unit sent out to all adults with brief admission contracts in Skåne in 2023.

BA unit	Number of letters
Lund	228
Malmö	147 (3 letters returned due to addressee unknown)
Helsingborg	76 (1 letter returned due to addressee unknown)
Kristianstad	28
Total	479

In addition to sending out letters we posted information about the study at hospital premises offering BA, in social media and in emails that were sent out to members through the help of the interest organization SHEDO (the Self-Harm and Eating Disorder Organization) in Skåne. We were open about the definition of a relative, in accordance with how the Swedish National Board of Health and Welfare defines relatives (Socialstyrelsen, 2014), stating that it could be any significant other, including family, partners, neighbours, personal assistants, and friends. Those interested in participating were encouraged to contact RL for more information about the study orally and in writing. Out of seventeen relatives who contacted RL regarding the study two were not available to participate and one considered themselves being too unstable for participation. Fourteen relatives participated in a focus group, of which six had experiences of BA provided at a mixed unit (Malmö) and eight had experiences of BA provided separately (Lund and Helsingborg). There were no participants from Kristianstad. The participating relatives were asked to fill out a questionnaire with background characteristics (Table 2). All 14 had close relationships and frequent contact with their relatives who had access to BA (which we hereafter refer to as *loved ones*).

**Table 2.** Participant characteristics according to information provided by participants based on questionnaire handed out to participants.

Gender (women/men)	10/4
Age, median (range)	56 (40–76)
Relationship to individual with BA contract, N	
Parent	8
Partner or spouse Parent-in-law	5 1
Duration of relationship in years, median (range)	24.5 (8–37)
Contact with individual with BA contract	
Daily	12
Every other day	2
Estimated duration of the loved ones’ BA contract, in years	
≤1 year	3
2–5 years	8
6–10 years	3
Estimated extent of the loved ones’ use of BA, total number of times, N	
2–5 times	4
6–10 times	4
11–20 times	3
More than 20 times	3

### Data generation

We performed three focus groups (lasting 83, 99, and 100 minutes) in face-to-face meetings in meeting rooms at hospital premises, moderated by RL and with JE or KL present as assistant moderators. The moderator’s role was to lead the focus groups by asking questions, follow-up questions, and initiating new areas for discussion when deemed appropriate. The assistant moderator’s role was to follow the discussion, helping the moderator by for example noting down content which could be followed up and directing questions to the whole group or to a specific person, depending on prior engagement, to make sure everyone had the chance to say what they wanted to say. We had prepared a semi-structured interview guide with open example questions, such as “What are your experiences of being a relative to a person with access to BA?” and “What does BA mean to you?”. The guide had been reviewed by representatives from SHEDO and the Swedish Partnership for Mental Health (Nationell Samverkan för Psykisk Hälsa; NSPH), a network of organizations for patients, users, and family carers in the field of mental health field, aiming to increase participation and influence quality assessments of health services (NSPH). Before beginning the focus groups, we served coffee, tea, and fruit and tried to create a calm and validating environment. Although some relatives talked more than others, all were actively participating. We encouraged relatives to talk to each other, by reacting and asking each other questions. Those who initially appeared to be more reserved were encouraged and invited to take part in the discussion through reflections or follow-up questions directed at them from moderators and other participants, for example in relation to prior statements, but also by allowing for moments of stillness and silence to create space for anyone who might have waited for an opportunity to share their thoughts and experiences.

### Data analysis

Since reflexive thematic analysis is a set-up which can be done in different ways, here follows a description of how we engaged in the process of the six analytical phases in accordance with the 15-point checklist for good thematic analysis as suggested by (Braun & Clarke, 2006).

*Familiarizing ourselves with the data* consisted of performing and transcribing focus groups, as well as taking notes on the side to capture thoughts about key words, ideas of themes and other things which we found to be noteworthy, while listening or reading through the data (the audio-recorded and transcribed focus groups). RL transcribed the interviews verbatim and included information on who said what in the transcripts by assigning each participant a unique letter in alphabetical order. In the process of generating codes from the data, RL (who worked from home) kept an extra notebook on the kitchen table to capture thoughts when away from the desk and listened to interviews repeatedly while walking the dog, sometimes stopping to record voice memos. We read and re-read the full transcriptions, highlighted words, and sections across the entire dataset, added analytic observations, and *generated initial codes* in Swedish from the interview transcripts. Notes on the side, voice memos, highlights in the transcripts and analytic observations, illustrations and reflections were used as support in the generation of initial codes and to collect thoughts on themes. Next RL worked through the initial codes and rewrote codes in English, which JE and KL gave written feedback on. We met and discussed, whereafter some of the codes were adjusted. We aimed to generate codes which described what was analytically interesting about the data, and to do it in a way which made it possible for us to remember the data behind the codes. As a result of this ambition some codes were latent (interpretive), but most codes remained semantic, staying close to the original data. We organized the data into a table with sections of text in one column and codes in English in another column.

For the *generation of themes*, we started the process of clustering codes after importing them into NVivo [QSR International Pty Ltd. (2021) NVivo, Version 14]. We began this process of searching for themes by inductively clustering very similar codes in many small groups. We read and grouped all the codes several times, sorting them based on different perspectives; first inductively based on different concepts relating the relatives’ experiences of BA (such as access to care and recovery) and then by trying out a more deductive way of organizing the codes, where we generated three broad buckets of codes simply referred to as (Liljedahl, Helleman, Daukantaitė, Westling, et al., 2017): the good stuff (Liljedahl, Helleman, Daukantaité, Westling, et al., 2017), the bad stuff and (Westling et al., 2019) the suggestions. These buckets of codes were then exported back into a Word-file and a finer, more detailed, and inductive grouping of codes was performed to *review, define, and name themes*. Hence, the generation of themes began and ended with inductive ways of engaging with the data in line with our chosen design, and in between those was a deductive element, an idea which came to us along the way when engaging with the data and which we found to be helpful while crafting the themes around relevant meaning-based concepts. This process we found to be a good way to get an overview of the content and to create a bit messy but still useful roadmap of our result (Table 3), which we revised and rearranged according to central meaning-based concepts across the three buckets of codes. We worked on the order to tell a story of the outcome of our analytical process and *produce the report*. The interaction among participants was incorporated into the analysis, exemplified below with quotes, all containing examples of interactions during the focus groups.

**Table 3.** Roadmap of results of relatives’ experiences of brief admission by self-referral where we used three “buckets of codes” to find patterns of shared meaning, underpinned by central meaning-based concepts, to generate rich themes.

Concept	The ‘Good stuff (positive/beneficial)	The ‘Bad stuff’ (struggles/challenges)	Suggestions to improve BA
ACCESS	We and our loved one’s experience safety and are relieved from rejection when seeking care. BA is easy access for our loved ones: just a phone call away. Our loved ones are relieved from hardships at the hated emergency care unit. Just having a BA contract is helpful. Complementary services and team-based work facilitates use of BA.	We experience limited or unpredictable access. Rejection when seeking BA leads to collapse. Prior trauma or ‘a speck of dust’ may overturn everything. Our loved ones are rejected at the emergency care unit, because BA is seen as a substitute. We fear losing BA if our loved ones don’t use it. Mixed unit issues: unit does not calm, and our loved ones feel less worthy.	Lower the threshold as much as possible. We can help map the hurdles to BA experienced by our loved ones. BA needs to grow to meet the demand of our loved ones but also needs to remain intimate.
INDEPENDENCE	BA is building ability and offers control to our loved ones. It is a learning process for our loved ones to get over the threshold to call about BA and use it when it is needed. BA means autonomy for our loved ones – and for us.	Our loved one’s experience rigidity from staff, such as being pressured to stay three full days. We struggle to release control. Our loved ones lose their ability to call when they need to – they struggle with timing BA. We force BA on our loved ones to be relieved from our burden of informal care.	Meet our loved ones halfway when they take the initiative to BA, even if BA is not available; offer a queue or call back. Adjust expectations according to our loved ones’ ability – especially when BA is new to them. Be patient – it takes time to learn BA. It may take over a year.
RECOVERY	We experience virtuous cycles of rest and recovery. BA means continuity, as our loved ones and we do not need to start over with emergency care and the staff is familiar. BA has a social value for our loved ones and reduces our relational strain. We experience ripple effects on family and work	BA is socially unfulfilling. Our loved ones experience supportive meetings with low quality. The staff at BA is young and inexperienced.	Introduce peer support for our loved ones on BA. Offer more inclusive activities at BA. Provide more education and support to us as relatives.

### Our approach to reflexiveness

Those of us who were actively involved in the data generation and analytic process (led by RL and supported by JE and KL) held reflective meetings on a regular basis throughout the analysis, beginning with reflecting on ourselves as persons and researchers, sharing self-reflectional texts with each other on qualitative approaches we had used in the past, our strengths, subjectivity, and theoretical positioning and how these might affect our interpretations. We reflected on the analytical process and compared it to previous methods of analyses we have worked with to actively decide on how to go about it. We allowed ourselves to learn along the way to stay open and flexible throughout the analytical process. After each focus group we jointly reflected on the content by taking some time to talk about it. During each phase of the interpretational process, RL took reflectional notes while listening, transcribing, and reading. Reflective notes consisted of text, voice memos and sketching of tables and figures, on computer as well as on paper.

### Ethical considerations

Before inclusion the participants were informed about the study orally and in writing and gave informed consent to participate. Focus groups were audio-recorded and stored in LUSEC (Lund University’s platform for storing, managing, analysing, and sharing personal data and other sensitive data securely) according to GDPR (General Data Protection Regulation in the European union). The Swedish Ethical Review Authority (Gothenburg, department 2, medicine) reviewed and approved the study (approval numbers 11 June 2019; 2019–02557; 16 June 2020; 2020–02098; 23 March 2023;2023–00582–02). After ethical approval we applied to the regional health authorities for approval to use names and addresses of those who had BA-contracts to send information letters. Letters were handled by a secretary at the clinic. The researchers had no access to personal data about those receiving letters or their relatives unless they themselves made contact.

## Results

We organized this results section beginning with a few paragraphs relating to how the participants described their lives in terms of being relatives to loved ones with self-harm, exemplified with data extracts. We believed this would enhance the understanding of the relatives’ experiences of BA. This is followed by a tabular overview of our generated themes and the central-based concepts. Thereafter follows more detailed descriptions of the themes.

Relative shared stories of the pain and powerlessness of life with a loved one with self-harming behaviour and suicidal thoughts. As loving, caring, and rewarding their relationships were, just as difficult was the life of being the stand-in; a full-time job without opportunity to ever relax fully—because what if you were suddenly needed. Relatives described their lives lived like that alongside managing the rest of the family and work. Some described how they were totally absorbed in the caregiving role and not able to work at all. Others shared stories of how taking care of a loved one could totally dominate an old-age retirement when you were the one who stayed after other family members had broken contact, fearing what will be when you are no longer there.

C: The worst is to feel so powerless, right? To not be able to do anything. When my child comes to me and says: “there is no point in me living. No one cares about me. Mom, you are the only one who loves me. (.) I might just as well die.” I mean, that powerlessness I feel as a mother in that moment. It is so painful.

From several: Yes. Mm. It is. It is so horrible.

C: It is. And it’s … I think it’s going to kill me. (laughs)

B: Yes, I feel the same way. (.) It is almost hard to … to … leave the house.

From several: Yes.

C: Yes. And I understand. You work, all of you work and …

B: No, I don’t work. It’s not possible to leave her alone either.

Issues such as own mental health problems, a busy job or having children with challenges such as ADHD or autism had to be put on hold when overshadowed by sudden life-threatening situations. The relatives talked about the fear of not being enough, acknowledging that they were already taking on too much. They said it could not be their responsibility to prevent a loved one from harming themselves, yet this was what they often did. They struggled trying to give their loved one’s space while seeing no other option than to keep checking on them. The relatives described being the first-aid support as well as the back-up at times when healthcare was unavailable or unable to help, a life full of worry, guilt, and relational strain.

H: It’s indescribable. There aren’t words for how horrific it is.

J: No.

G: No, like, your situation must be very difficult.

H: (.) One time. (.) she showered for seven hours. So she was lying. like a little sparrow in the corner and we couldn’t get her out of the shower. Like, she still felt dirty. And I had to call a doctor on duty. He couldn’t solve it. (.) I finally got her out and into the car. And we arrived at the emergency room. Totally wrecked. But there was no room. So we drove to a. little village nearby and spent the night in the car. (.) Because, like, we were afraid to go back home to that environment.

The relatives appreciated being able to participate in our study and connect with others in similar situations, saying it was emotional and strengthening to hear about different experiences. Some relatives said that they had chosen to participate because they were afraid that the concept of BA might be discontinued.

From the relatives’ stories we generated three themes based on central meaning-based concepts (Table 4). The themes are described below and woven together with data extracts, organized in an imagined chronological order related to entry into BA, learnings, and outcomes.

**Table 4.** Central meaning-based concepts and themes on relatives’ experiences of BA.

Central meaning-based concepts	Themes *What were the relatives trying to tell us?*
Access	A low threshold to a safe back-up is crucial and obstacles may easily break faith
Independence	Trust in their ability with care and respect
Recovery	The rest and relational recovery that we all get are needed and invaluable.

The relatives described meanings and effects of BA for the relatives themselves as well as for their loved ones. The relatives often took the stance of their loved ones, telling us about their loved one’s feelings and experiences, based on the relatives’ own perceptions.

### A low threshold to a safe back-up is crucial and obstacles may easily break faith

The lowered threshold to care with BA was described as a relief after earlier fights for entry into psychiatric care. Relatives described negative experiences and doubts stemming from experiences of an obstructive healthcare system.

Access to BA had *provided safety and relief* from hardships, the latter often linked to traumatic events at the emergency care unit (ECU). Access to BA replaced the fear of rejection with predictability. Turning to the ECU was fearful waiting without being welcome, admissions of uncertain length and alive-keeping storage—which could be crucial at times but not healing. With a BA contract access to help was only a phone call away. BA did not require an unknown physician’s approval or humiliating processes of sharing their lives story again and again. BA had spared the relatives from having to “sell in” how ill their loved ones were. BA meant access to pre-emergency help with less drama.

K: What I find so very, very positive is that you don’t have to go to the emergency room. Because I find that to be such a giant threshold. To go there. And then you have to sit there and wait. And then you have to be assessed. And then they need to see if there is possibly any room for you. You are relieved from that entire process, which I find to be an enormous advantage.

J: I completely agree.

G: Yes, if I get a phone call like “I am staying at BA, I’m on BA” she says. Then I think: Yes. Oh, how nice.

Relatives described how *having a BA contract meant safety* even in between admissions. Their loved ones talked a lot about BA also when they had not used it much. Relatives described BA as an open offer, while other healthcare was expressed as being full of closed doors and limitations.

D: Sometimes safety is just knowing that you have the possibility. The right to use BA. Then you don’t have to use it. It helps just knowing. Because then the person feels a little better. And can continue at home.

F: Yes, that’s true.

E: Yes, that’s kind of how it is. Easier.

A: Yes, but I got a little scared of that now that he hasn’t been there/for a while/. Because I thought about that. If he is not there. Do they then think that he might be healthy enough to manage on his own and that he does not need it, so to speak? And then I thought: I don’t want that. He might not be there. He might never go there again. But it’s like …

E: the safety of just knowing, yes.

A: … the oxazepam in your pocket. (Laughs) Being allowed to have something. So that you can.

Relatives described BA as *part of a team-based complementary service* alongside DBT (Dialectical Behavior Therapy) and FACT (Flexible Assertive Community Treatment), unburdening emergency care by being enough. Where one type of service stopped another continued, altogether forming a coherent safety net for a group who otherwise easily lost context, according to the relatives.

N: I also think that collaboration is important. That there are several who can catch her, in a way. So, I think that the combination with FACT (.)/and/BA creates that net. They so easily slip through the net. Like stopping having friends. And everything just keeps getting worse. (.) Having a sort of net around you that is connected. That’s important.

Interviewer: Is BA part of that net?

N: FACT and BA is a collaboration. Like you said (turning to M): they go to BA and write the contract. And/FACT/knows if you are admitted. Like, somehow, they are always informed. So that they know.

M: Like, [my son] can also set up appointments. That they are here (meaning the hospital) instead. So that if he is on BA/the appointments/will come here instead of home. And have their meetings or whatever we call it. And I also think that is good. It’s like … It makes it a little easier. Because before he thought that “no, I cannot go to BA because the psychologist or the nurse (.) [are coming]”. But that works just fine.

Relatives shared several aspects related to access to BA which they perceived as problematic. These were a mix of direct access issues and psychological challenges increasing the reluctancy to call for BA. The relatives shared stories of *BA not being available when needed* as all beds were occupied. which could cause stress and worry, especially if this coincided with them not being available as back-up. Relatives emphasized that the number of beds and staff were too few to meet the demand for BA. They advised adapting BA to the demand with extra beds when needed, while emphasizing the importance of BA being kept intimate with little staff turnover to keep the threshold low. Although rejection was not perceived as a problem for some (“available tomorrow if not today”) other relatives said that a “no” when someone calls for BA was a risky failure which could be *perceived as taking away the last option*. Trauma from prior rejections at the ECU were awakened when loved ones called and failed to get a bed at BA. This could make loved ones collapse again and not call back for BA for months.

N: If you call at eight sharp it is not [too late] but ten minutes past eight it is.

M: Yes, that’s really how it is. So. I don’t know if there maybe should be more available beds. But just in the beginning, the first half of the month, then maybe. Because [my son] has received a ’no’ more than half of the times he has called [about BA]. That it is fully occupied. Ehm. And for him it will take maybe two months before he calls back.

Relatives shared experiences of how ECU staff—implicitly or explicitly—had referred to having *a BA contract as a legitimate criterion for not being provided emergency care*. Relatives said that suggesting BA as a substitute was a way for ECU staff to relieve their conscience during busy times, which was understandable but seen as unfair and dangerous.

Although some relatives said that their loved ones were content *using BA at wards mixed with regular admissions*, others described the atmosphere at a mixed ward to be triggering and a threshold for loved ones to use BA. Relatives said that they found it odd that admitting to BA could mean sharing rooms and bathrooms with suicidal strangers, some of which were there unwillingly and at their worst. It had happened that staff had implied that patients who were worse off had to be moved because their loved ones had called about BA, making them feel uncomfortable and guilty. Relatives also said that their loved ones had perceived limited rights to care or consultation at the mixed ward, coming home to the relative totally exhausted. Circumstances like these had contributed to a resistance to call again about BA.

Relatives emphasized *the importance of identifying and addressing hurdles to seek and use BA*. Mapping of hurdles could be done by carefully asking those with a BA contract if and why they had not used BA when needed. They advised to also ask BA-users about permission to ask relatives to map the hurdles to call about BA. Relatives said that it was especially important to take extra care early on when BA was new to a person, for example by giving new BA-users the option to only stay for a few hours to get accustomed to the ward. This could be enough to sense a break from an approaching crisis and lower the threshold to use BA again.

### Trust in their ability with care and respect

*The process of learning to use BA* was a slow but rewarding process according to the relatives. They said that their loved ones had gradually learned self-management, that BA had been perceived as unpredictable to begin with, when leaving the safety of home, not knowing if BA would help. Relatives themselves had sometimes struggled to let go of control, finding it hard to accept that the ward would not call if their loved one would discharge themselves from BA. Learning to back off was described as a balancing act between “better safe than sorry” and keeping a good relation. BA required that their loved ones had the will to seek help and in turn required the relatives to back off. The relatives said that for their loved ones to come somewhat to terms with their wellbeing required patience and time.

L: Even if the home environment is safe. (.) But in an environment like [BA]. Then you don’t have to think about [cleaning and cooking]. Instead you can. focus on yourself. Yes.

M: Yes, like my son (.) I believe it took him a year before he tried it out for the first time. (.) Because he thought it would be like being at that other ward where he had been. Ehh … And thank God it wasn’t. Now he is really happy about [BA] being available.

N: My daughter says the same. That she thought it would be like being admitted. Because she has been admitted to compulsory care and everything else. She has said several times that [BA] is completely different. I mean, it’s the ward next door but that the staff and the entire approach is completely different. And that [BA] gives you a positive sense, but that [other admission] on the other hand gives you a negative sense. It’s not fun at all.

M: No.

L: I also believe that it is, yea, a threshold in itself that. Just the environment in itself. And my wife has also wished: cannot BA have its own ward?

Relatives talked about the value of *BA offering control and building ability* in terms of helping their loved ones to do something on their own and getting to know themselves. Getting a BA contract was confirmation of having a problem and being considered to have the capability to take care of it. As their loved ones practiced and learned to ask for and receive help through BA, they were also able to let their relatives help them, which the relatives described with joy and fulfilment. The life of the relative had up until BA been characterized by a desperation of wanting to help—not as an involuntary and poor form of healthcare substitute but as a partner or family member. The relatives described being able to help in ways they themselves preferred and could cope with, such as cleaning their loved one’s kitchen or taking care of the children or the cat while their loved ones were getting back on their feet with BA. The relatives said that BA had meant relief from constant involvement and attachment. The process of learning to use BA was described as a collaboration and joint effort between the relatives and their loved ones. BA had helped in making both parties more independent and equal adults who could soberly reason about their needs.

E: Like, it’s not as sharp as going to the emergency room.

F: No, exactly.

E: And then it is so much easier to talk about. And reason around on an entirely different level. And that helps a lot. In general, I mean. It does. Both in the relationship and. Yea. It helps create the sense of safety that they need.

A: (.) Because “Do you need to rest?” or “Do you need to be admitted?” Where “admitted” feels like being related to some sort of straitjacket. Because you are locked up. Locked. So …

B: I think it is a little like an open entry to healthcare

Relatives emphasized the importance of their loved ones *being able to choose rest and care actively and independently*, because only they themselves could know when they needed help. Negotiating a contract meant involvement in care adapted to their wants and needs. Having access to BA had made it easier to carry on at home, giving a sense of safety for both the relatives and their loved ones. Free will and ability to act according to their choice meant that their loved ones had used BA in different ways, sometimes prophylactically, sometimes for remedy, and sometimes for the sake of their relatives, when they thought that the relatives needed to rest. The relatives said that access to BA facilitated their loved ones’ ability to be responsive to their relative’s needs, making their loved ones happy and proud.

Relatives described *challenges counteracting BA’s aim to support independence*. They gave examples of how loved ones had been approached by staff with rigidity. Examples included being forced to leave their room for hours, having activities limited by rules related to risks of self-harm, or having the three-day concept imposed on them. The relatives acknowledged that they themselves in a desperate need for recovery, sometimes had pressured a loved one to stay the full three days. Relatives said that being encouraged to initially take one day at a time had lowered the threshold and contributed to BA feeling free and flexible rather than scary and heavy.

Relatives shared experiences of how loved ones had *lost their ability to act independently* when needing BA the most. They said that this was linked to making telephone contact being too demanding and requiring feeling good. An example was a loved one feeling like BA was completely lost when the possibility to send text messages to the BA ward was abolished due to changes rules on secrecy. Relatives described that what may be perceived as a detail to healthcare could mean that their loved ones entirely lost the right to act independently.

To support their loved ones’ ability to grow independent relatives said that in some cases it *would be reasonable for healthcare to meet halfway*. Acknowledging that loved ones need to feel that BA is on their initiative, relatives still meant that it could well be reasonable not to force loved ones to have to revisit the threshold the next day as this might rule out BA as an option. This could for example be done by calling those back who had called about BA when there were no available beds. This would be especially important if it happened one of the first times someone called about BA, considering how difficult it could be to gather the courage to call in the first place. Relatives said that it was about having an optimal level of challenge—not too easy, nor too difficult—to gradually build competence among their loved ones.

Relatives said that it could be relevant to consider having a waiting list on BA, and even to provide the possibility to plan for and prebook BA, for example once a year, on special dates where their loved ones knew ill health would escalate, such as a date where an especially traumatic event had happened, or to offer loved ones the power to offer their relatives a chance to get away.

M: Sometimes he has said “but go do something for the weekend” and then he calls ten minutes past eight on Friday morning. And then “there was no room for me. So you have to stay at home.” Yes of course we will stay at home. Of course. It doesn’t mat… But that disappointment he. he has in that. Not being able to. Because he knows that he needs us. He could… If he is feeling bad he cannot be alone. (.)

L: If you get a “no” today, can’t they at the same time say “yes, but we will get you a spot for tomorrow”? So that they don’t have to, like … revisit that threshold the next day. But they get that message already then.

N: That would probably be good too, yes.

L: Yes.

M: Be put in a queue.

L: Yes, exactly

### The rest and relational recovery that we get are needed and invaluable

Relatives emphasized the power of BA to offer their loved ones and themselves time and space to do what they needed to do—apart from each other to meet anew again.

Relatives described BA in terms of *virtuous cycles of rest and recovery* for everyone involved. Knowing where their loved ones were and that they would be ok made the relatives able to relax, let go of control and responsibility as less was on them. They did not have to chase their loved ones on the phone, and they dared to sleep. It meant some time off for both parties to see each other again when feeling better and rested. They said that BA was an amazing relief at times when relatives were totally wrecked, a breather from the ill health they were constantly living with.

M: BA is in a way also for the relatives, even though you don’t go there yourself. But it feels like it’s the only thing that you have as a relative.

Interviewer: Even though you aren’t there?

N: Yes (Laughs)

M: Or actually maybe thanks to [us not being there]. Yes, no, but it is.

Interviewer: What do you think about that? Like, that [BA] can have that function?

M: I think it is so wonderful. Because. we live with this all the time, always. With this, like, ill health. So it’s a break. It’s soo important.

Relatives described BA as a combination of psychological and physiological recovery for their loved ones. BA had contributed to breaking negative patterns of thoughts and offered practical help when being out of energy, enabling loved ones to regain routines for eating and sleeping before they have hit the bottom. At the same time relatives said that their loved ones, safe and empowered with BA, were feeling less guilty. BA offered loved ones a healing stillness where they could let go of despairing thoughts and slow down. Food was served and they could read and craft without stress. It provided a loved one who was constantly at home a valuable and safe change of environment.

H: She can’t stand herself. Can’t cope with her own troublesome thoughts. Because everything she doesn’t want to think about; that is what she thinks about. Yes. (.) And then it’s just wonderful to be allowed to call and get that change of environment. I mean that’s. Yes. Like, it’s worth so much. Yes.

J: It’s a safe environment also, then?

H: Yes. I mean, absolutely. And of course. You struggle with yourself and you’re on your knees anyway sometimes. And then. And these three days. Oh! It’s just like (laughs) this. That’s how you feel. So that you can recover.

BA was described as a completely different set-up for refuelling and a less dramatic step compared to emergency care, being described as traumatic admissions where loved ones were let out just to quickly return. Relatives said that BA was a healing middle-way that their loved ones could control. For the relatives BA meant freedom and opportunity to finally focus on themselves and their needs. An example was rehabilitating their own mental health problems which had been set aside and prolonged. Or just be happier at work when not getting calls as often and having to leave early due to emergencies.

Relatives emphasized the *social and relational value of BA*, helping them to draw a line between being a close and engaged relative and providing care. They described how they could use BA as part of an agreement with their loved ones. Self-referral to BA could be used by the loved one when the relative could not fulfill their need to be listened to. The relative would take care of the kids and the home as long as the partner with access to BA took responsibility for their care. Relatives said that arrangements like these reduced the relational strain. Knowing where to turn earlier, loved ones were spared from involving their relatives. Relatives were spared from having to seek care with their loved ones and relieved from situations such as hearing about suicidal plans at the ECU. Relatives described their loved ones as being very responsive to the relatives’ needs and wanting to do what they could, knowing that relatives were happy when the needs of their loved ones were fulfilled. The ability for loved ones to take early action made both parties more functional partners to each other, being less tired, guilty, and ashamed.

D: Because they are able to go there before they. hit rock bottom. By cutting it there they become a more well-functioning partner. And thereby [we have] a more well-functioning relationship as well.

E: Yes. I totally agree with that.

C: Yes, I agree. It’s just positive. (.)

E: Yes, but I also think about the thing with shame. That they don’t want to … involve more people and so on. That it helps a lot that they are able to take the initiative. That affects the relationship a lot. That they don’t end up in a place where they are ashamed because they have …

D: … burdened us.

E: Burdened us, yes exactly.

According to relatives, BA gave their loved ones a valuable social context in all the loneliness of mental illness. Talking to staff was an important reason to seek BA. Meeting the same professional staff on BA meant coming back to an ongoing conversation with someone who knew their loved ones’ story. The relatives said that this was extremely valuable in terms of being helpful, predictable, and familiar. On the other hand, *lack of conversations or young inexperienced staff* giving unsolicited advice could be dismantling to their loved ones. Relatives shared stories of staff managing to tempt a reserved loved one to take part in an activity, experience a positive meal or discuss pictures of their pets. Having a little fun in all the not so fun and something new to talk about were small things that made a big difference for their loved ones—and therefore also for the relatives themselves.

Relatives *recommended providing more social support* to both them and their loved ones. They suggested introducing peer support on BA arguing that this might provide additional valuable contact to someone who can understand. They also suggested increasing the number of inclusive and voluntary activities on BA such as walking or painting. As for themselves, relatives said that they knew they were relieving healthcare significantly but felt like they were lacking psychoeducation. They wished for more targeted education and support, suggesting arrangements on regional level since they had found local support initiatives to be too light.

## Discussion

Relatives described BA as offering cycles of rest and recovery for both them and their loved ones. They said that BA gave their loved ones the opportunity to take control and fulfil psychological and physiological needs. For the relatives themselves BA was a possibility to live more freely and make plans of their own, even if only for one night. For this discussion, we want to highlight the aspect of relational recovery linked to access and outcomes of BA, shared by our participating relatives. We also wish to emphasize the importance of organization of care in relation to BA and its implications for practice.

### Relational recovery

The change in and importance of relationship dynamics which the relatives talked about during the focus groups pointed to reciprocal processes of recovery, stemming from the fact that loved ones had been granted trust to use BA without being questioned. In everyday life BA could be part of open practical discussions between relatives and their loved ones about how to use BA, just as discussions on other matters such as childcare or managing cooking and cleaning. Qualitative studies on BA have indicated similar experiences among BA-users, describing experiences of increased ability to stay grounded in their social environment, maintain social roles and reduce the strain on close relationships (Helleman et al., 2018; Lindkvist, Westling, Liljedahl, et al., 2021).

Relatives shared experiences of how their loved ones had been able to use BA not only for themselves but also to unburden them as relatives, who were otherwise 24/7 standbys with tremendous distress and need for rest, recognized from other research on relatives to individuals with self-harm and suicidal behaviour (Lantto et al., 2023; Marshall et al., 2023; Mughal et al., 2022). BA helped directly by fulfilling both parties’ personal needs for recovery and indirectly; when the relative felt better, the loved one with access to BA also felt better—and the other way around. The ability for loved ones to experience how BA unburdened their relatives, was described by relatives as strengthening and giving their loved ones a rare opportunity for independence and empowerment. Their loved ones—a group of individuals who were otherwise always receivers of care—were able to give something to their close relatives, making both parties more equal. As such, BA provided a new and crucial form om healing, according to relatives, pointing at recovery as being an interdependent social process between individuals and their context, which may be referred to as relational recovery (Price-Robertson et al., 2017). However, relational recovery was dependent on loved ones being able to trust that BA was available when and as needed. Since trust was easily broken, according to the relatives, it takes caution to avoid or make sure that a negative experience in relation to BA (such as an experience awakening trauma from prior care or perceptions of not being listened to) does not overturn all the positive.

With BA, relatives needed to adapt to a new role, which may have been more of a struggle among parents (which over half of our participating relatives were), sometimes having a hard time letting go of being the constant guardian while wanting to give their loved one’s rooms to be the adults that they were. Similar experiences of difficulties to adjust to a new role are recognized from research on parents’ experiences of BA for teenagers who self-harm at risk for suicide (Lantto et al., 2023).

### Organization of care

Several factors related to the organization of care were raised in this study, for example related to devoting enough beds, the significance of the environment, and integration of care. The safety of knowing that a loved one could self-refer to BA for care and support when needed was highly valued by relatives in our study, as seen also in other studies (Hultsjö, Rosenlund, et al., 2023; Socialstyrelsen). However, the perception of availability could easily change. Trust was just as crucial as easily broken. Repeated failed trials to get BA raised the threshold to call again. The issue of beds not being available when needed has been seen before in relation to relatives experiences of BA in Sweden, affecting their ability to trust BA (Hultsjö, Rosenlund, et al., 2023). There has been a general increase in overcrowding in Swedish psychiatry during the last years (Regioner SKo), which may be linked to the relatives’ experiences of BA being used as a substitute by emergency care staff, which is serious given that that BA is not an emergency admission and therefore should not affect access to other care, including emergency care (Liljedahl, Helleman, Daukantaitė, Westling, et al., 2017).

Relatives stressed the importance of continuity and a calm environment at BA. Mixing BA with emergency admission and sharing bedrooms or bathrooms with other patients was described by relatives as stressful to their loved ones. Single patient rooms with private bathrooms have been described as the most importance factor to reduce stress and aggression in psychiatric inpatient wards while shared rooms may disturb privacy and rest (Ulrich et al., 2018).

Relatives highlighted beneficial aspects of integration of care, appreciating interprofessional team-based collaboration, where BA was integrated with other services such as FACT, together creating a network of services which they perceived as facilitating and responsible health care. These benefits of FACT contributing to a less fragmented system has been experienced before by health care providers (Trane et al., 2021).

### Implications for clinical practice

Based on the perspective of relatives, our study points towards opportunities to strengthen implementation as well as potential improvements and adaptations of BA.

With respect to implementation of BA increased information and communication about access to BA (for example that access is not affected by non-usage) to users and relatives could lower the threshold to seeking BA. From the perspective of health care mapping the demand for BA, for example by keeping statistics on rejections, and adjusting the supply, possibly with more beds during “high demand” periods might contribute to maintain the predictability of BA as intended. Furthermore, measures to maintain the BA concept through for example education and follow-up are continuously needed to avoid adrift from adherence. Supervision of the desired BA approach to support staff working at BA wards may prevent experiences of rigidity and support autonomy and recovery among users.

Table 5 provides a brief overview of some of the suggestions brought forward by the relatives for healthcare to consider meeting needs in relation to access, independence and recovery.

**Table 5.** Needs in relation to Brief Admission and relatives’ suggestions for improvement.

Need	Relatives’ suggestions to healthcare on how to further improve BA
To lower the threshold	• Map general and individual hurdles to seeking BA by asking users and relatives about it.
To support development of autonomy	• Consider possibilities to adjust expectations on users’ independence in relation to individual capacity, especially for those being new to BA. • Inform relatives about the value of encouraging loved ones to use BA.
To support rest and recovery	• Consider introducing peer support and more voluntary group activities.

Suggestions for improving the BA concept include mapping of individual hurdles to lower the threshold to BA, such as carefully asking those with a BA contract, and their relatives, if and why BA has not been used when needed. Writing these hurdles into the contract and discussing strategies on how to handle them might support users. Relatives may be invited to contribute, which our participants mentioned. Giving relatives opportunity to be involved in the BA concept and give them a more central role in care might increase benefits of BA according to prior research (Hultsjö, Appelfeldt, et al., 2023; Hultsjö, Rosenlund, et al., 2023). Recent Swedish standards highlights the need for making better use of the competence of relatives by integrating relatives’ perspective in healthcare (Socialstyrelsen, 2023). Meeting those with a BA-contract halfway and “rewarding” contact regarding BA, for example by calling those back who were denied a place at BA if no beds were available, might be beneficial. In general, expectations on autonomy need to be adjusted in relation to individual need and capacity, especially for those new to BA, so that the level of challenge is optimal to build competence. It might be an idea to inform relatives about the value of encouraging loved ones to use BA but without pressuring them. Lastly, relatives recommended introducing peer support on BA and offer more inclusive voluntary group activities to further enhance the social value of BA.

For the relatives themselves they asked for targeted psychoeducation and regional support groups for relatives, as local initiatives such as supportive meetings or education were not perceived as relevant for their specific situations. Interactions with others facing similar difficulties can enable relatives to individuals with suicidal behaviour to gain new perspectives and experience reduced distress. This was mentioned in the focus groups and is also concluded in prior research (Juel et al., 2021).

### Methodological reflections

We chose to apply the approach of reflexive thematic analysis due to its flexibility and reflexivity. To us it appeared to be an agreeable and well-adapted approach when studying the complexity of humanity and human experiences. We found it to make sense because it builds on consciousness about the method and us working with it. The focus groups provided an opportunity for generating rich data through interaction (Peek & Fothergill, 2009). The participating relatives were engaged and interacted with each other more than with us researchers, asking each other questions and reflecting on each other’s statements. Possibly, this was the result of the relatives’ need to meet others in similar situations and share experiences. Although we at times guided the focus groups by bringing up new topics, most of the questions we had prepared were brought up by the relatives on their own initiative. Consequently, significant parts of the discussions happened without much interference from us researchers and resulted in richness and depth in data.

Our study builds on a standardized and evidence-based model of psychiatric self-admission (Westling et al., 2019). The participating relatives in our study were linked to three of the four clinics in a large uptake area with many BA-users at both separate and mixed wards for BA. The participants included both men and women at different ages and relationships to those using BA. They all had very close contact with their loved ones with access to BA.

We used passive recruitment strategies through targeted mailings to all individuals with a BA-contract in the region, information at the BA units, emails to members of SHEDO and social media. We were not able to make direct contact to relatives to adults with access to BA as this was considered too invasive in relation to ethics. Still, with close to 500 letters being sent out we had expected more participants than fourteen in this study. RL received a phone call from one individual with a BA-contract who said they did not have anyone to give the letter to, which might have been the case among additional others. Loneliness and social isolation have been mentioned by individuals with access to BA (Lindkvist, Westling, Liljedahl, et al., 2021). One relative called RL to say that they appreciated the opportunity to participate in our study but that they were too worn down to be able to participate. This appeared as not surprising given the descriptions by the participants in our study of the burden of being a relative to a person with self-harm and suicidal ideation.

We saw it as a strength that we were three individuals with different experiences and backgrounds involved in the analysis. All three had prior experience of qualitative research. Two of us had done prior research on BA in the Skåne region and one of us had experience of doing research on a similar model in Stockholm. We believe this added to our reflections throughout the analysis. We aspired to be transparent by providing detailed descriptions of our procedures and demonstrating our subjective positions. Using personal pronouns in the methods section was one way for us to point out our active roles in generating the results.

## Conclusion

Our results point to BA as an approach which supports and creates value through relational recovery. The possibility to use BA for self-referral according to personal needs may, according to our results, provide positive effects on quality of life for both users and relatives. Involving relatives more may help healthcare to take caution to details which are important to users, and which may overturn positive effects of BA. Several aspects of BA were, according to relatives, implying a crisis-management intervention in line with core competences of healthcare, such as team-based work and person-centred care, and a holistic approach to self-harm care. Implementation may be strengthened by measures such as supervision and follow-up of BA staff and education of emergency care staff as well as increased resources to make BA accessible without limiting access to emergency care. Mapping hurdles to BA, especially among beginners, and support through peers and targeted psychoeducation may improve recovery for BA-users and their relatives.

## Data Availability

Focus groups supporting the findings of this study are not publicly available due to privacy.
